# Sustainable Packaging Design for Molded Expanded Polystyrene Cushion

**DOI:** 10.3390/ma16041723

**Published:** 2023-02-19

**Authors:** Normah Kassim, Shayfull Zamree Abd Rahim, Wan Abd Rahman Assyahid Wan Ibrahim, Norshah Afizi Shuaib, Irfan Abd Rahim, Norizah Abd Karim, Andrei Victor Sandu, Maria Pop, Aurel Mihail Titu, Katarzyna Błoch, Marcin Nabiałek

**Affiliations:** 1 Faculty of Mechanical Engineering and Technology, University Malaysia Perlis, Arau 02600, Perlis, Malaysia; 2 Center of Excellence Geopolymer and Green Technology (CEGeoGTech), Universiti Malaysia Perlis, Kangar 01000, Perlis, Malaysia; 3 Faculty of Material Science and Engineering, Gheorghe Asachi Technical University of Iasi, 41 D. Mangeron St., 700050 Iasi, Romania; 4 Faculty of Civil Engineering, Technical University of Cluj Napoca, Constantin Daicoviciu No 15, 40020 Cluj Napoca, Romania; 5 Industrial Engineering and Management Department, Faculty of Engineering, “Lucian Blaga” University of Sibiu, 10 Victoriei Street, 550024 Sibiu, Romania; 6 Faculty of Mechanical Engineering and Computer Science, Częstochowa University of Technology, 42-201 Częstochowa, Poland

**Keywords:** closed-cell foam, drop test, finite element analysis, design parameter, protective packaging

## Abstract

A molded expanded polystyrene (EPS) cushion is a flexible, closed-cell foam that can be molded to fit any packing application and is effective at absorbing shock. However, the packaging waste of EPS cushions causes pollution to landfills and the environment. Despite being known to cause pollution, this sustainable packaging actually has the potential to reduce this environmental pollution because of its reusability. Therefore, the objective of this study is to identify the accurate design parameter that can be emphasized in producing a sustainable design of EPS cushion packaging. An experimental method of drop testing and design simulation analysis was conducted. The effectiveness of the design parameters was also verified. Based on the results, there are four main elements that necessitate careful consideration: rib positioning, EPS cushion thickness, package layout, and packing size. These parameter findings make a significant contribution to sustainable design, where these elements were integrated directly to reduce and reuse packaging material. Thus, it has been concluded that 48 percent of the development cost of the cushion was decreased, 25 percent of mold modification time was significantly saved, and 27 percent of carbon dioxide (CO_2_) reduction was identified. The findings also aided in the development of productive packaging design, in which these design elements were beneficial to reduce environmental impact. These findings had a significant impact on the manufacturing industry in terms of the economics and time of the molded expanded polystyrene packaging development.

## 1. Introduction

Packaging design is an important element in the packaging value chain along the product distribution process because it will determine the effectiveness of the protected product [1,2]. The packaging design also functions as a crucial component in the packaging value chain because it determines the materials, manufacturing process, and environmentally friendly options for disposal [3,4]. The molded expanded polystyrene (EPS) cushion serves as protective packaging to prevent a product from shock and vibration impacts [4,5,6]. This packaging application has been extensively utilized across many industries, not only in food packaging, but also for all kinds of fast-moving consumer goods and household and electronic appliances [7,8,9,10,11]. Presently, the most popular polymer-based cushioning for home appliances is polystyrene (PS) foam [12]. Polystyrene foam is classified into two types: expanded rigid open-cell, which produces loose-fill foam, and expanded flexible closed-cell, which can be molded into a variety of shapes with a normal density range from 11 to 32 kg/m^3^ [13,14,15,16]. Due to its high impact resistance, its ultralightweight, durable, low thermal conductivity, and its ability to be molded into any shape or size, this rigid foam has been used as a shock absorber (inserts) in a variety of packaging appliances [7,12,17,18,19,20,21].

Today’s manufacturing industry is rapidly evolving to provide competitive products [6,22]. This product development is also improving in parallel with new technologies nowadays [23]. However, global production will continue wreaking havoc on society and the environment if these tendencies are not promptly addressed [24]. Hence, in the long run, packaging designers should consider designs that are sustainable and environmentally friendly to reduce pollution, prioritize environmental protection in design, and provide a design concept that adheres to ecological ethics [23]. Concurrently, the development of packaging designs involves a variety of disciplines such as history, science, engineering, economics, and social responsibility, all of which are heavily reliant on the engineer’s knowledge and experience [25,26].

Packaging design has given a comprehensive surge, considering multiple factors appropriate to the complexity of sustainability challenges [27]. Thereby, packaging design for sustainability exemplifies the fact that it is made up through multiple elements: the natural environment, society, and economic performance [28]. Continuous improvement is necessary for packaging sustainability, and even minor modifications may lead to substantial gains for the environment (material), economy (cost), and society (manufacture) [29,30].

Hence, in the development phase, designers’ decisions play a key role in ensuring that environmental repercussions are reduced. Approximately 80% of a product’s environmental impact is defined at the design stages of the product development process [31]. Designers are in charge of specifying the material selection, how raw materials are processed or manufactured, and how products are packaged, distributed, used, and eventually disposed of [11]. Hence, this paper presents the investigation of design parameters that must be prioritized in producing a sustainable design of EPS cushion packaging. Currently, many literature reviews have emphasized the importance of EPS cushions as protection to ensure product safety [1,15,32,33,34]. Product packaging influences recycling behavior; thus, recyclability of the packaging should be considered a precious value of the packaging, allowing multidisciplinary research due to the complexity of the recycling behavior, between user waste management and the technical part of the system [35]. So, this study investigates the relationship between packaging design and environmental sustainability, discovering a positive impact on the industrial community and economy, particularly in the home appliance-manufacturing sector. The concept of sustainable design development entails using natural resources by protecting environmental values, reducing the use of resources, and elevating the quality of life [36]. Therefore, sustainable design is defined as environmentally responsible product design and development that integrates a product life-cycle perspective and incorporates work, culture, and organizational skill approaches. In terms of applying sustainable packaging design, the evaluation criteria applied to products are reducing material usage and diversity, reducing energy consumption, reusing the product, and reducing the weight and volume of the product [37]. Monteiro et. al. found that 58 percent of respondents believe that sustainable design is extremely important for the packaging industry [38].

Thus, destructive testing and a preliminary finite element analysis simulation are carried out to determine the main parameter of sustainable design of EPS cushion packaging. Cushion reliability impact is used to manage risks and understand how they affect the quality of the product, allowing design considerations to be made [39], whereby the acceleration response and strain histories of simulation results are correlated with experimental measurements [40,41]. The explicit method is preferred over other methods to simulate the drop tests [42]. Drop testing and the finite element analysis method are employed in the development process to evaluate design flaws and to see potential product damage caused by packaging design weakness (potential to be reused and reduced). The detailed results of both methods are then compared. The results will be used to produce a new molded expanded polystyrene packaging.

## 2. Methods

**Figure 1:**
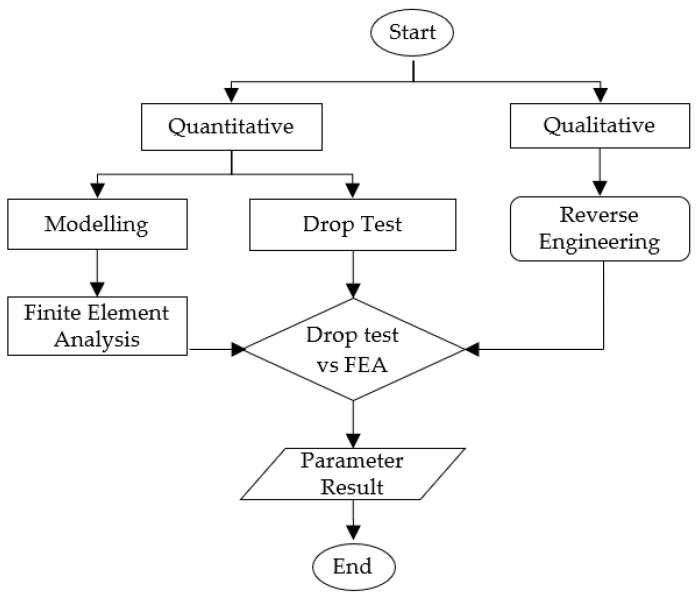
Flowchart of design and development method.

This study used two particular quantitative methods (see Figure 1), which are drop-test verification and finite element analysis validation. The drop test is performed to detect serious damage and to determine the dependability of the packaging design in order to protect products of varying weights or sizes [2]. Time and cost are major constraints, where the empirical approach is applied repeatedly [43]. Provisionally, the drop test was carried out in accordance with the standard procedure ASTMD5276-19 and met the requirements of ISO Standards 2206:1987 and 2248:1985. Furthermore, the finite element analysis (FEA) approach was carried out to compare the findings, and the effectiveness of using explicit analysis was demonstrated. The analysis was simulated using ANSYS software version 2019. Finally, the design parameters for sustainable cushion packaging were identified.

### 2.1. Drop Test

**Figure 2:**
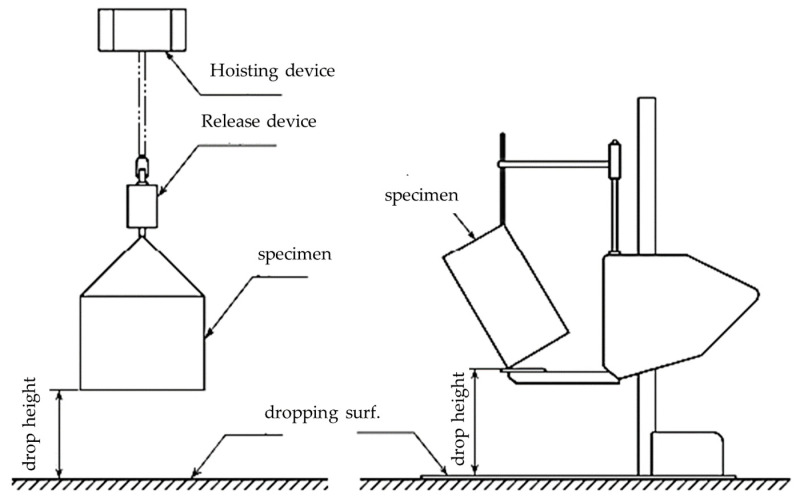
Drop-test apparatus [41].

The design verification of cushion packaging was conducted using a drop-test apparatus (illustrated in Figure 2). The results were compared to FEA simulation analysis to identify the area of defects and possible impacts on products such as cracks, dents, or breaks. There are five designs of EPS cushion packaging that have been tested from different heights, depending on gross weight and packaging surface, as performed using the test apparatus mentioned in JIS Z0212 standard [41] and also as referenced in ASTM D5276 [16].

**Figure 3:**
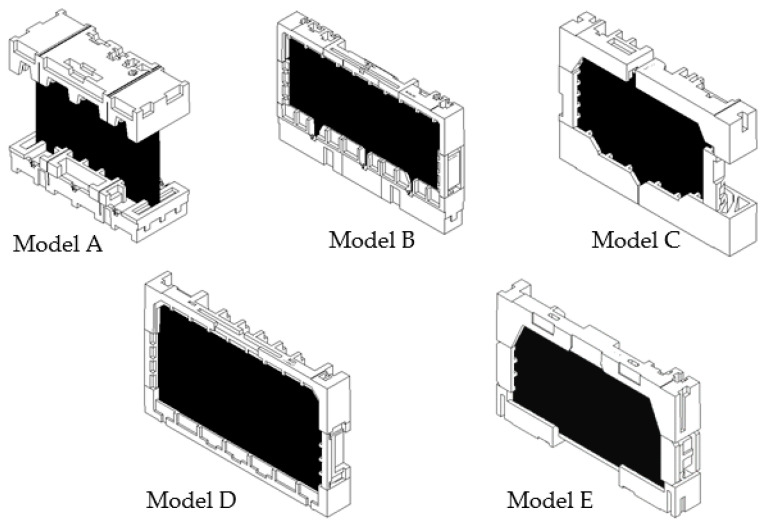
Molded EPS packaging design models.

The different sizes of packaging with similar appearance designs (see Table 1) were selected to study the drop impact against cushion packaging, as shown in Figure 3. This is the primary factor used to determine the most important design parameters to incorporate. These five types of packaging design were also chosen due to the variety of container loading efficiency (CLE) quantities, which contributes to comprehensive comparison and validates the main parameter of sustainable packaging design. These primary parameters will be followed in the design and manufacture of sustainable molded EPS packaging.

**Figure 4:**
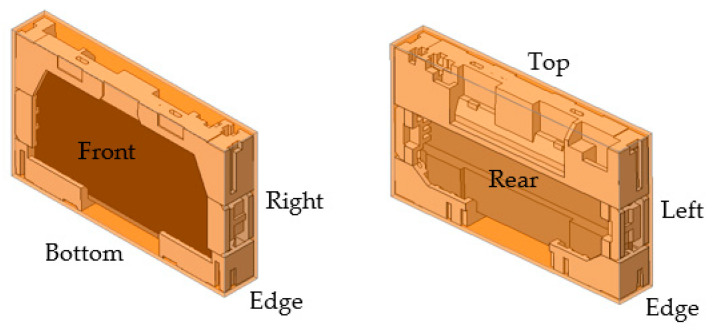
View of package surfaces.

Drop test was performed on pilot package sample. The complete assembly of molded cushion packaging and the product itself are used to assess the possibility of damage during transportation or handling processes. The drop height is specified in Table 2. The surfaces for drop test are shown in Figure 4. The drop-test packaging must then be successful in order to protect the product from deformation defects in the ±2.0 mm range and avoid defects on the product, such as cracks, bezel damage, scratches, light leakage from the screen, and component damages.

The allowed height range should be ±2% or ±10 mm, whichever is greater.

Table 3 shows the test sequences that can be partially eliminated, depending on the type of packaged freight. Then, the test sequence can be modified, but prior agreement with the test requester is required. Furthermore, the chosen corners and edges to be tested will be the wake ones because corner drop testing helps determine the ability of the contents inside the packaging system to withstand rough handling [44,45,46].

**Figure 5:**
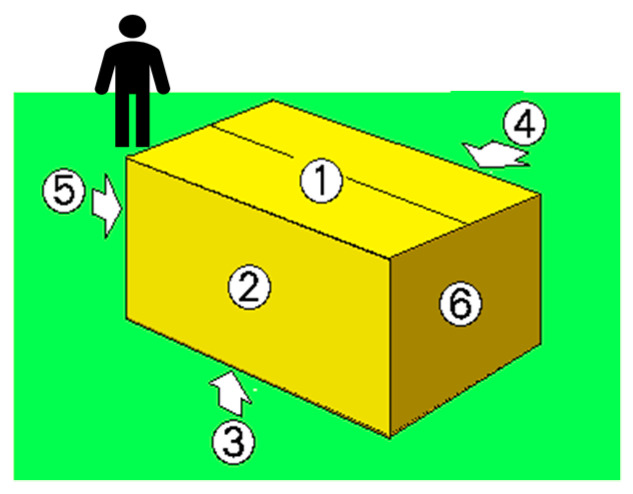
Drop-test placement if face direction are known [41].

The package must be placed in the position intended for transportation. However, if it is known, it must be placed vertically on the observer’s right. When the package is positioned with one side facing the observer, the upper surface of the package is identified as No. 1, the side on the observer’s right as No. 2, the bottom as No. 3, the surface on the observer’s left as No. 4, the nearest side (front) as No. 5, and the side farthest away or rear as No. 6 (see Figure 5) [45].

**Figure 6:**
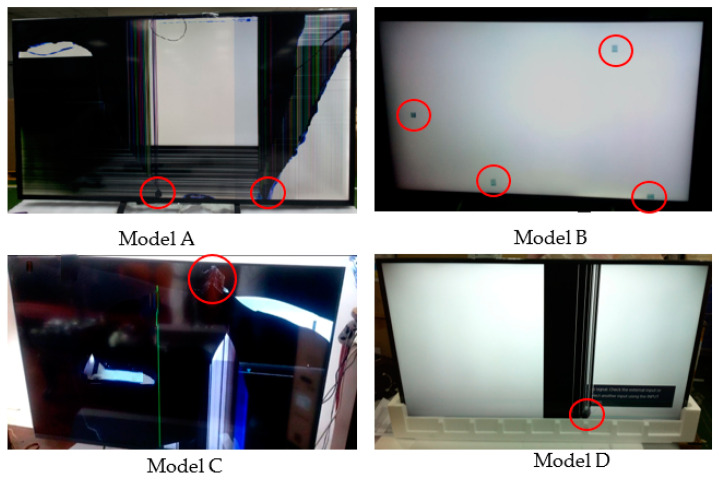
Drop test result.

**Figure 7:**
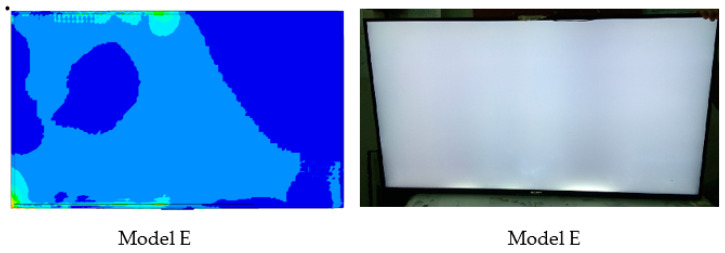
Drop-test results.

The results of the drop test are as follows: Three EPS cushion packaging models encountered a serious design failure on the screen panel, as shown in Figure 6, while two other models faced a minor design failure on models B and E, as shown in Figure 7. The findings of the summary found that the drop-test results of models A, C, and D show that the screen panel is cracked in several different places. Model A demonstrated that almost the entire panel was cracked, beginning at the bottom of the product. For model D, the insufficient cushion thickness puts pressure on the bezel, which directly causes screen damage. When it was decided to use two pieces of packaging, the starting point of the screen panel crack of model C was at the top of the product. This area was not protected by cushion packaging, as shown in Figure 3. Next, a drop test for model B found a scratch defect on the screen surface of the product. Moreover, a light leakage defect occurred on the bottom packaging of model E. This illumination leak was caused by the design rib of cushion at the bottom, and this flaw also led to an unclear panel quality of the product while in use.

**Figure 8:**
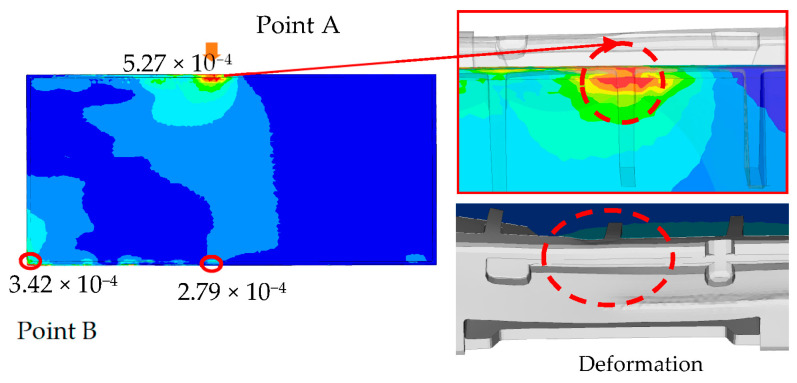
Impact of EPS cushion design on product performance.

**Figure 9:**
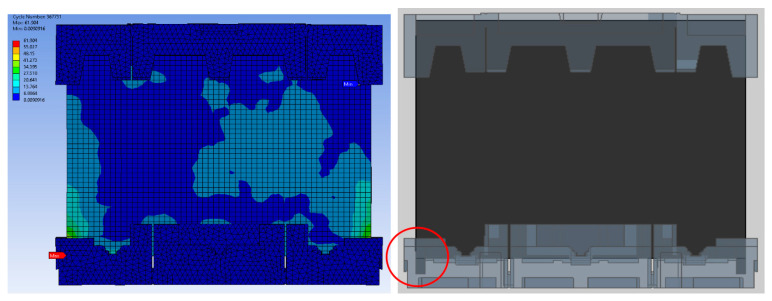
Analysis result of model A.

**Figure 10:**
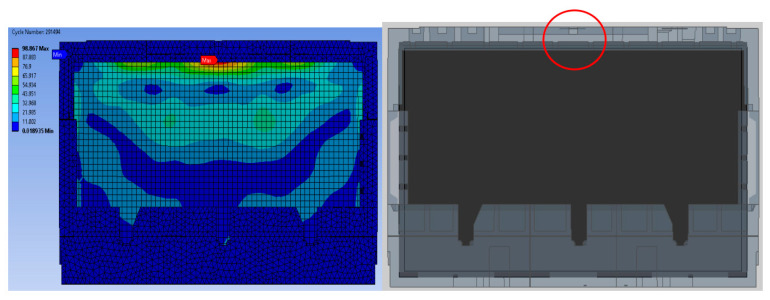
Analysis result of model B.

**Figure 11:**
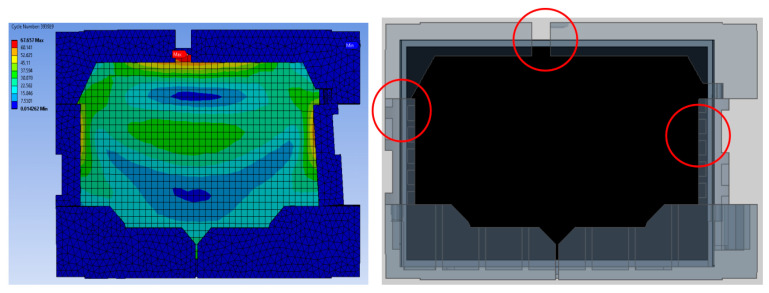
Analysis result of model C.

**Figure 12:**
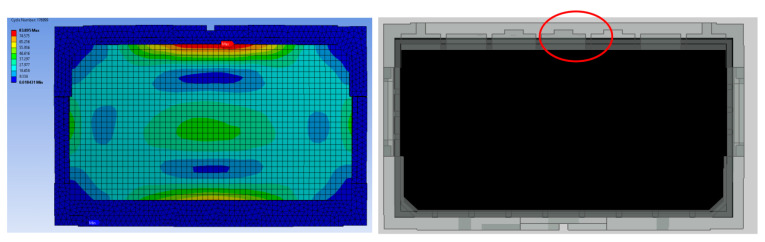
Analysis result of model D.

**Figure 13:**
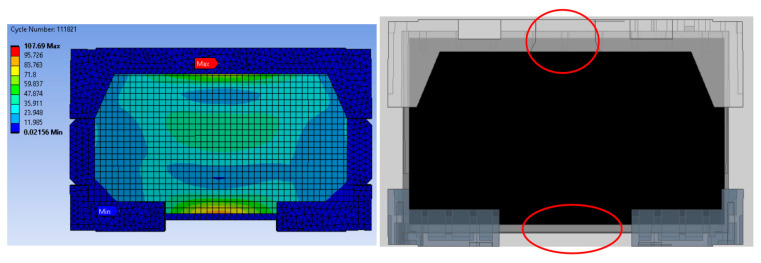
Analysis result of model E.

The root cause of failure was discovered to be caused by factors such as rib positioning, thickness, and cushion shape itself due to the disparate levels of expertise among packaging-design engineers. Therefore, finite element analysis is performed to compare the design parameters in detail, and the most critical design parameters are identified from the critical area in research findings (see Figure 8, Figure 9, Figure 10, Figure 11, Figure 12 and Figure 13).

### 2.2. Finite Element Analysis

Expanded polystyrene foam is the best rigid substance to use as protective packaging for household appliances, but its sustainability is in doubt. Therefore, its reuse and reducing waste were the biggest contributions from packaging-design engineers [47]. Additionally, this study also agreed that the analysis is one of the effective testing methods that should be carried out during the product development process [5,10,12,42,48]. However, it is important to follow the cushion design parameters in order of importance. At the same time, analysis of structural appliances was also considered, such as positioning of electronic board, screen panel, speaker bracket, etc. [2,5,10]. So, the material properties of products and packaging were obtained, as shown in Table 4, for further analysis. The packaging engineer’s expertise will also be at an equivalent standard. Packaging design weaknesses are overcome, and cushion waste is reduced.

Explicit dynamic analysis was used in this finite element analysis. It is set that the product’s packaging is dropped with a gravity parameter of 9806.6 mm/s and the floor is in a fixed rigid setting. Product damage is predicted. Improvements in sustainable design were implemented at an early stage of the product development cycle. The maximum stress and deformation direction are used as a reference in the analysis result to predict the critical area that would have design failure. The FEA analysis was compared to the results of the drop tests.

Figure 8 shows the impact of EPS cushion design on product performance, where the critical areas are located at point A and point B. The current design (design A) was modified before the molding tool development stage. To reduce the impact on the product’s front surface, the thickness of the EPS cushion was reduced. High strain is thought to occur as a result of the pushing effect of the set locally, where the cushion ribs are located.

### 2.3. Analysis Result of Drop Test

The comparison of packaging design model analysis results in numerical maximum stress values. The maximum stress area was also compared, as shown in Figure 9 and Figure 13, implying that design failure is possible. The maximum stress predicted during the drop test is highly mesh-size-dependent; there has to be a balance in choosing the mesh size, such that it yields accurate results and is computationally efficient. Contrary to this study, the optimum mesh size for five models of packaging design is chosen to be 20 mm, with skewness settings used to show that the mesh structure was close to its ideal form. The analysis results revealed that the maximum impact result on the cushion indicated that each of the packages required design improvement before molding development process.

**Figure 14:**
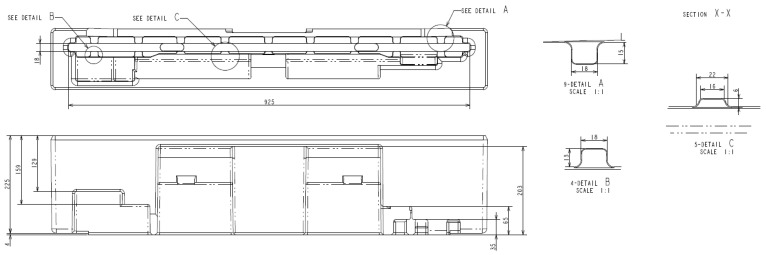
Dimension of rib.

**Figure 15:**
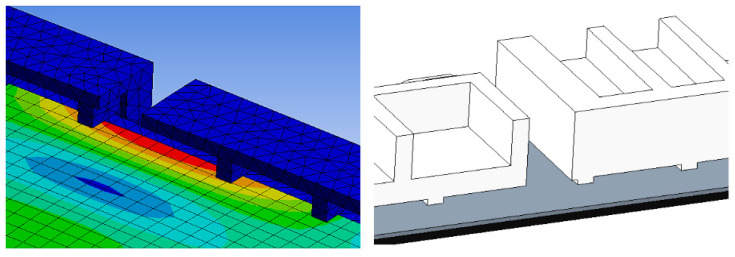
FEA result.

The numerical value of the maximum stress was discovered to be marginally different for each model of packaging that has been studied. The highest value for model A was found on the bottom side of the packaging, while models B and D were found on the rear side of the cushion packaging, as stated in Table 5. Lastly, the maximum stress of model E and C cushion packaging was found on the front and left sides, respectively. This maximum stress was used to predict the possible areas of defects that exist, without going through the mold modification design repeatedly. As a result of the analysis, all of these cushion packages required design improvements. Firstly, the improvement design for model A focused on cushion thickness in order to reduce cushion stiffness and to withstand the hazards’ impacts. Models B and D were both modified in terms of rib dimension, and uniform tolerance between the rib, screen panel, and rear cover was ensured. The product’s accessory positioning must then be considered in order to determine the optimal rib dimensions (see Figure 14). Finally, models E and C demonstrated a proclivity for screen and clip components to be damaged. As shown in Figure 15, modifying the position of the cushion layout is required due to a lack of support that caused panel screen damage. Although the cushion packaging layout can be determined, packaging size must also be evaluated and calculated, especially when measuring container loading efficiency.

Differences in analysis results are caused by the cushion packaging’s productive design. The high strain was close to the most critical component: the screen. This outcome demonstrates that it is likely to damage the screen and clip internal product components. Due to the lack of support on the front and back cushions, as shown in Figure 15, it is necessary to alter the position of the cushion layout on the upper cushion.

In general, the results of all analysis models are influenced by the EPS cushion design parameters as outstanding protective packaging. However, the primary important factors need to be ensured to produce sustainable cushion packaging. The analysis results show that the packaging design of models C and E are seen to be similar, but the cushion layout and length of bottom inner cushion are different. This difference in cushion layout has produced the same analysis results, where the possibility of damage is shown on the critical component, which is the screen panel, as illustrated in Figure 9 and Figure 13.

On the other hand, the design considerations of molded packaging for models D and B are the same in terms of cushion layout design, dimensions, and thickness, even though the product size is different. Both models show the same analysis result, which is the emphasis on the position and dimension of ribs, predominantly on the top of the cushion packaging. However, because the installation of pre-existing accessory components must be taken into account, the cushion packaging model A that has been made differs greatly from all four models. Cushion thickness and rib positioning, moreover, have been identified as the main design factors to emphasize. The comparison results of destructive testing and finite element analysis are discussed below.

**Figure 16:**
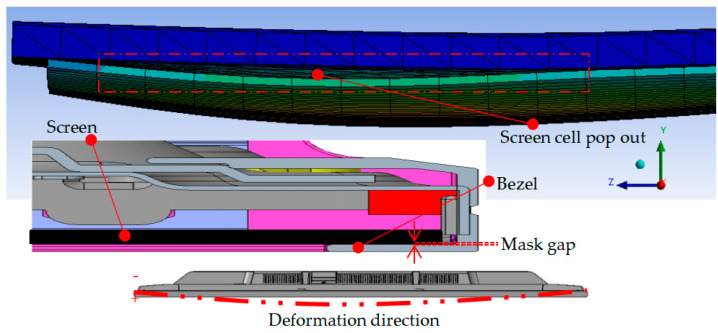
Design failure.

A damaged or cracked screen panel is a major problem that has been identified. This indirectly causes product deformation defects, as shown in Figure 16. It shows that the defect happened due to high stress impact at the edges of the cushion packaging, which initially intended to hold the product. Unfortunately, it has produced a cell pop-out defect on the screen panel. The number of ribs and their position have thus been identified as the primary design parameter. At the same time, it is vital to ensure that the mask gap between screen and bezel must be within the specs of the product standard.

The analysis of design failure concludes that there is a lack of cushion packaging toughness to resist shock loads or impact. The design reliability is lower than expected due to several factors. First, the position of rib was placed on a sensitive area, such as on the middle chassis or on the clip of the panel board. This clip is placed in the front of the screen panel, which is used as a holder for each layer of the screen projector. This clip must avoid being exposed to any pressure or impact. Secondly, there is insufficient rib strength in order to absorb the impact, and the wall thickness of cushion is less than 10 mm (minimum thickness). In fact, the strength of cushion is influenced by the position, height, and thickness of ribs. In addition, the packaging weight also affects cushion stiffness. Therefore, the cushion weight must be reduced, and the design needs to be improvised if necessary. Minor consideration, such as tolerance between packages and goods, must be checked by considering the material types of protection bags. Commonly, the thickness of protection bags ranges from 0.02 mm to 0.4 mm.

In shear modulus (G), the shear stress (Ʈ) is directly proportional to shear strain (ɣ) as expressed in Equation (1) [49]:

G = Ʈ/Ɣ(1)

The ratio of normal stress to normal strain within the elastic limit is known as young’s modulus of elasticity (E) [13,21,25]. It is a material property and remains constant for a given material. When a material is subjected to shearing load, the ratio of shear stress induced to the corresponding shear strain is a constant within the elastic limit, and this constant is known as the rigidity modulus (G). Poisson’s ratio (ν) is another material property which is the ratio of the lateral strain to longitudinal strain when loaded within the elastic limit [50]. The relationship between the two elastic constants is shown as follows.

E = 2G(1 + ν)(2)

where E is the Young’s modulus, G is the rigidity modulus, and ν is the Poisson’s ratio.

**Figure 17:**
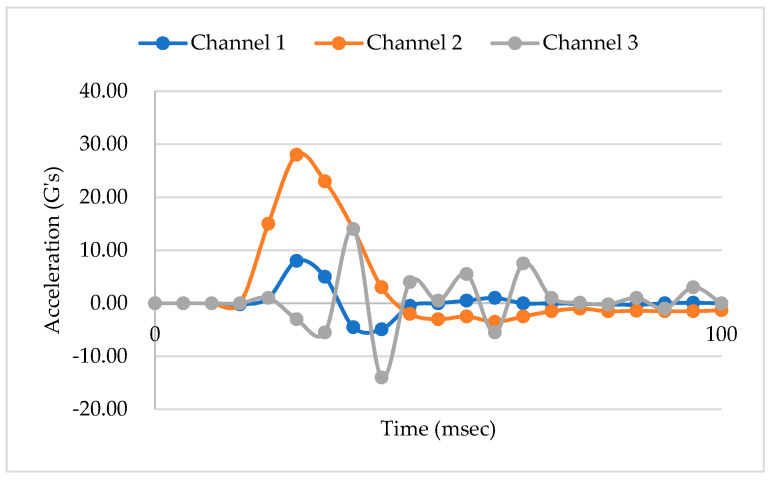
G’s value result from drop test.

Practically, the rigidity modulus (G) value can also be defined through the experimental drop test. The machine used in the drop test was connected to a PC. All parameters were controlled and monitored by the PC. The value was discovered from sensor detector that are attached together inside appliances and packaging, as shown in Figure 17. The highest value among the channels would be selected as the shear modulus of rigidity (G) result. Product fragility is standardly determined by an actual drop test as provided in ASTM D3332. Shock testing can help determine a product or packaging system’s level of fragility by measuring the amount of input acceleration required to damage the product’s function or cosmetics. It is often measured in terms of fragility [25,43,46].

**Figure 18:**
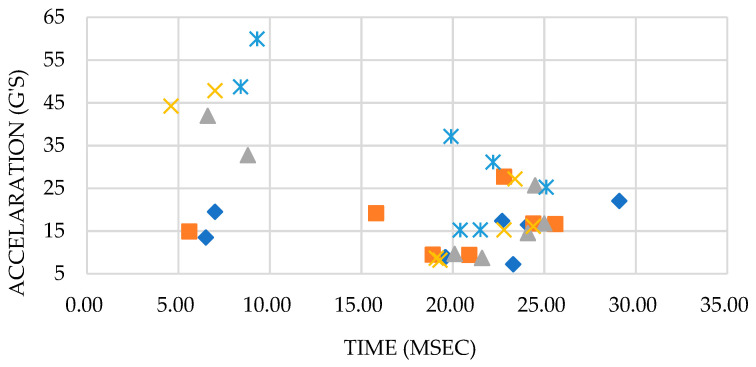
Acceleration (G’S) vs. Time (msec).

However, the comparison result shows that the maximum modulus of rigidity that is obtained through the destructive test is in the range of 8 G’s to 30 G’s per surface of each following model, as illustrated in Figure 18. The results show that the peak acceleration is dominated by the cushion packaging model E, while the lowest value is obtained from the cushion packaging design model A. Therefore, it has been proven that the cushion design will affect the fragility of EPS cushion packaging. The design of packaging model A is more complex than other cushion packaging designs.

## 3. Results and Discussions

**Figure 19:**
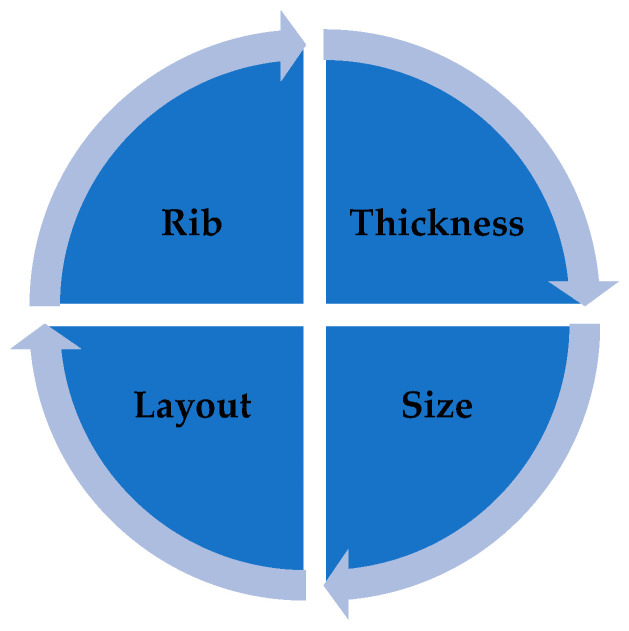
Parameter of cushion design.

The string of this study has assumed that sustainable EPS cushion design should consider the weight and shape of the cushion (design optimization). Logically, the effectiveness of cushion packaging reliability is based on the complexity of the design or the tendency to reduce overall packaging material usage, which is in line with previous studies [5,10,16,17]. This study has proven that there are four main design parameters that need to be emphasized in designing cushion packaging, starting from the conceptual design phase. At once, the design for sustainability will be able to be implemented in product development, especially in the manufacturing industry. The main parameters to be considered in producing the sustainable design of EPS cushion packaging are graphically shown Figure 19.

Generally, waste from EPS cushions can be recycled in many ways once it comes to the end of its life. The choice of recycling methods is based on technology, environmental, or economic factors. The authors’ views are similar to that of the study by Muralikrishna and Manickam (2017), where the interpreted life-cycle assessment is a cradle-to-grave analysis technique to assess environmental impacts associated with all the stages of a product’s life: from raw material extraction through to material processing, manufacture, distribution, and use [51]. Accordingly, this study has discovered the main design parameters in the development process of sustainable EPS cushion packaging. Furthermore, the impact of CO_2_ reduction that will be employed in the manufacturing sector is disclosed [52], and the waste-management options are evaluated from an economic perspective [53].

Knowledge of specific sustainable product design principles, such as designing for repair/reuse/remanufacture/recycling, is widely recognized as a vital skill and is supported by an earlier study [54,55]. The importance of understanding sustainable product design techniques, as well as how to select and implement the most appropriate ones based on the design challenge, was emphasized [24]. In this current study, an EPS waste hierarchy is introduced as a waste-management priority order: (1) preparing for reducing and reuse, and (2) design optimization, besides evaluating the waste-management options from an economic perspective [53]. Reusable packaging is the first option that designers should try to implement, if possible, because it does not mandate costs for recycling processing and remanufacturing. It is clear that this approach fits the circular economy concept [56].

A combination of the life-cycle assessment (LCA) and life-cycle cost (LCC) analysis was significant [57], allowing for cost savings, a reduction in manufacturing time, and a reduction in carbon dioxide emissions due to the sustainable design. Previous research has indicated that, in order to achieve sustainability, certain markers must be used, and the variables that affect the state of economic, social, and environmental problems must be controlled [58,59].

The ISO 14,040 rules state that an LCA study consists of four stages: defining goal and scope; inventory analysis; impact assessment; and interpretation. This guideline framework defines how much of a product’s life cycle is engaged in sustainable design [60]. On the other hand, LCC allows for the identification of potential cost drivers and cost savings for a product or service throughout its entire life cycle (a project or a product from acquisition, installation, operation, and maintenance to the final disposal of the raw material) [61]. The majority of the contributions are focused on direct applications to product development and improvement. Due to this reason, this design parameter has been influencing the reduction in cushion packaging cost and time, as shown in Equations (3) and (4).

(3) $$\mathrm{Cost}\;\mathrm{saving}\;\left(\%\right)=\frac{\mathrm{Total}\;\mathrm{development}\;\mathrm{cost}-\mathrm{total}\;\mathrm{modification}\;\mathrm{cost}}{\mathrm{Total}\;\mathrm{development}\;\mathrm{cost}}\times 100\%$$

(4) $$\mathrm{Time}\;\mathrm{saving}\;\left(\%\right)=\frac{\mathrm{Modification}\;\mathrm{time}\;\mathrm{of}\;\mathrm{mold}}{\mathrm{Total}\;\mathrm{development}\;\mathrm{time}}\times 100\%$$

**Figure 20:**
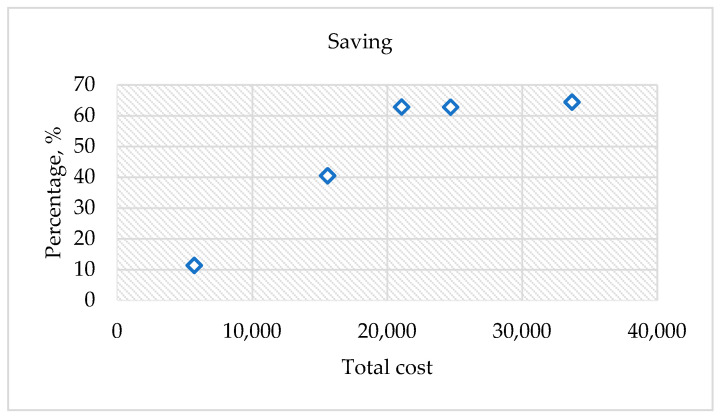
Percentage cost to be saved.

**Figure 21:**
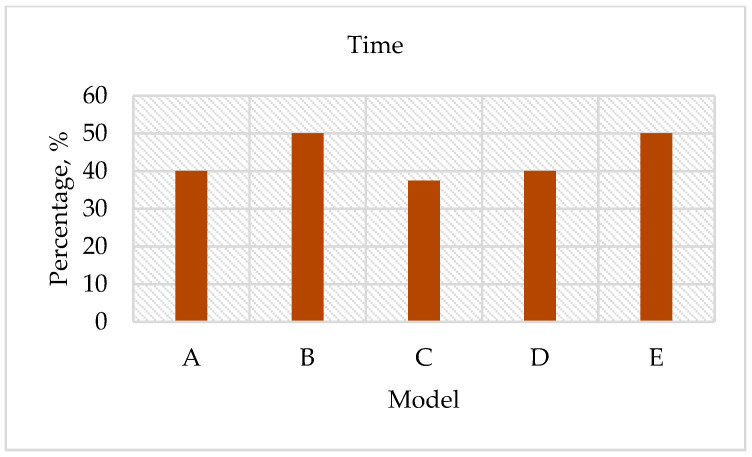
Modification time reduction.

**Figure 22:**
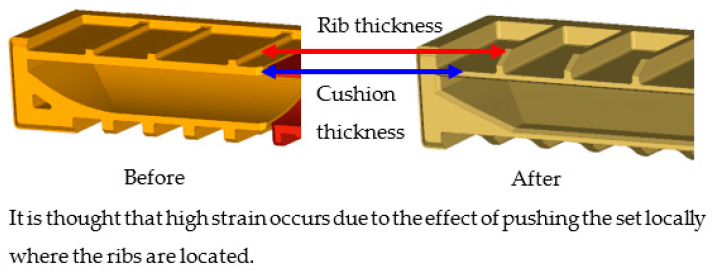
Improvement finding using simulation analysis.

**Figure 23:**
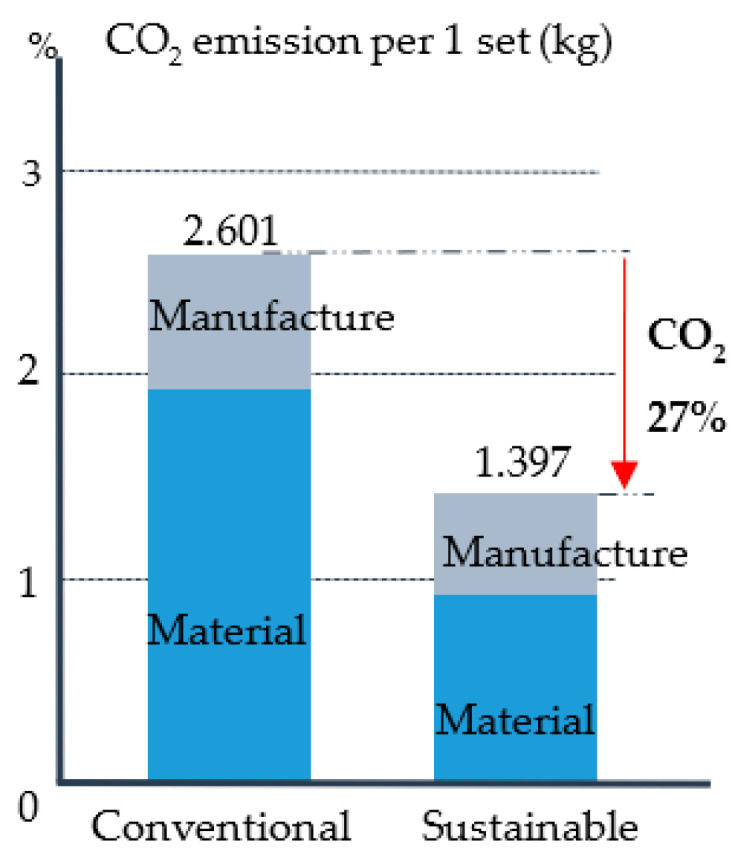
Carbon dioxide (CO_2_) reduction.

**Figure 24:**
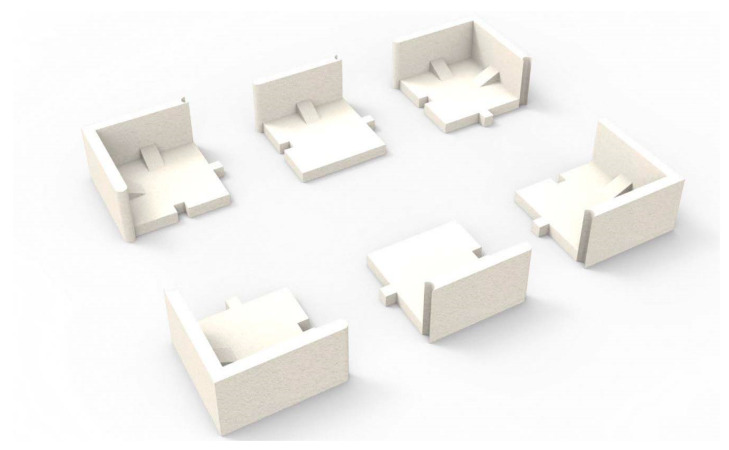
Design of dovetail technique.

**Figure 25:**
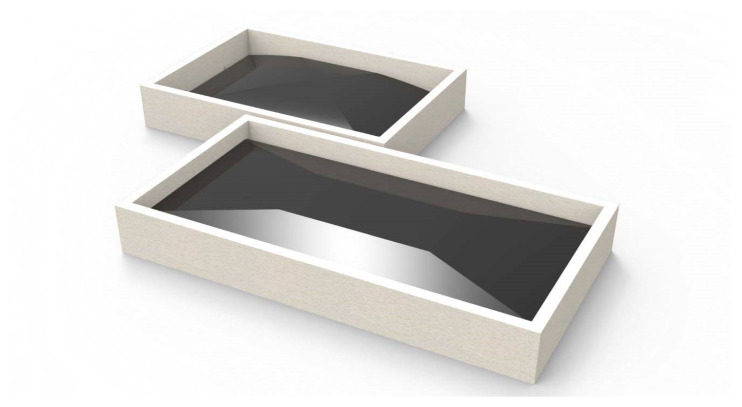
Multi-size application for EPS cushion packaging.

Finally, this paper was found to be beneficial in saving 48% of the development cost; had a 25% modification time reduction; and estimated a 27% carbon dioxide (CO_2_) reduction. The summary of the findings has been summed up into the below accomplishments:

- Costs were saved by 48% of the total cost of the cushion packaging development (see Figure 20). These average cost savings were identified from the five cushion packaging designs. This percentage will increase drastically if improvements in the cushion packaging designs can be made. At the same time, the implementation of sustainable design needs to be applied in the early stages of the product development life cycle. Four main parameters are emphasized without going through the trial-and-error process. So, it is able to reduce the EPS materials use in the product development cycle. Furthermore, it is proven that the relationship between cost development and effective design will contribute to sustainable EPS cushion packaging, as well as reduce EPS waste by avoiding the trial-and-error methods in the design validation process.
  Significant cost differences are found in the development of sustainable EPS cushion packaging. Modification costs are higher when improving the design if it is based on conventional practices. However, by incorporating these discoveries into a sustainable design (using the design parameters and reducing the use of EPS cushion), the massive amount of modification costs can be well organized.
- In the meantime, the average mold modification time was also saved by 43.5%, as shown in Figure 21, because repeated modification and retest verification can be avoided. This is because the possibility of design defects can be identified using the finite element analysis method as shown in Figure 22. Improvements will be made before the development of the molding tools for EPS cushion packing. This study was successful in validating the use of the analysis method in creating a new cushion design with sustainability elements. The results of the analysis, as shown in Figure 7, previously made it clear that cushion rib and thickness are the main parameters to be considered in order to reduce EPS waste disposal.
- The most significant finding is the reduction of carbon dioxide (CO_2_) released from sustainable design method. It is estimated that improvements in packaging design, material, and multiple functional uses have cut CO_2_ emissions by as much as 27%. The value of CO_2_ emission per one set model (kg) was calculated. The comparison of the reduction is shown in Figure 23.
- Additionally, it was discovered that the grafting method, which entails using a dovetail technique in rib cushion design, is used in optimization design to input a new parameter (refer Figure 24). This design technique can also be interchanged to fit multiple sizes of products (see Figure 25) and together manage the cushion layout or align a product orientation position.

**Figure 26:**
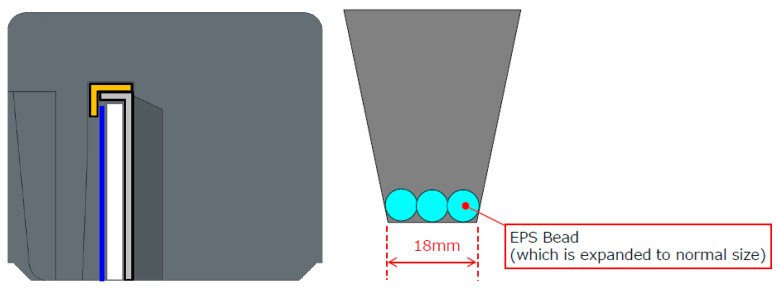
Cushion rib.

The rib dimensions determined by the dovetailed technique are 40 mm × 20 mm × 20 mm, with a 25-degree slant. However, the detailed rib design consideration for new molded cushion packaging is depicted in Figure 26. If a cushion thickness of 18 mm is chosen, the cushion bead is expanded to normal size and the cushion’s shape, or strength becomes stable.

## 4. Conclusions

The intention of this study is to emphasize the design parameters that should be applied in cushion-packaging testing using destructive testing, finite element analysis, or risk analysis. The findings have a great potential to minimize the pollution impact on the environment and save development costs and materials, as well as realize an EPS cushion design sustainably. It would also be beneficial to the manufacturing industry in managing the EPS cushion waste. Therefore, the tendency for simulation analysis usage in industry is accepted to enhance designing process efficiency and improve skills in bringing forth sustainable protective packaging for electrical and electronic appliances. Hence, it can be concluded that:

- The significant parameters of EPS cushion design increase the packaging reliability.
- The sustainable design can be implemented, and packaging design can be optimized through the manufacturing process and cost.
- Finite element analysis of the cushion design was a great idea for analyzing possible defects caused by design failure before the molding tool development begins.
- Optimization design through the reuse and reduction of EPS cushion usage contributed to increased design sustainability.
- The challenge in this finding is that every packaging engineer must change their current design practice and analyze the packaging design prior to the development phase.

More research is needed to identify the biodegradable raw material or mixed composite based on these discovery parameters, especially for the molded expanded polystyrene cushion packaging of electrical and electronic appliances.

## Figures and Tables

**Table 1:** Packaging size.

Model	Package Size (L × W × H)	Types of Rib	Rib Position
A	1684 × 506 × 1080	Inner and Outer	Symmetric
B	1332 × 170 × 827	Inner and Outer	Symmetric and Asymmetric
C	1292 × 177 × 764	Inner	Symmetric and Asymmetric
D	1187 × 158 × 732	Inner and Outer	Symmetric
E	1016 × 152 × 625	Inner	Symmetric and Asymmetric

**Table 2:** Drop-test height.

		Height, h (cm) of Surface Dropped			
Model	Gross Weight, W (kg)	Bottom	Front Rear	Right Left	Corner Edges
A	57	30	25	25	25
B	23	40	36	36	36
C	19	50	40	40	36
D	14	55	45	45	36
E	12	55	45	45	36

**Table 3:** Drop sequence and number of times [41].

Sequence	Portion to Be Impacted	Test Times
1	Bottom adjacent corners Ex. Corner 2-3-5	1
2	Side adjacent edges Ex. Edge 3-5	1
3	Bottom-side face edge Ex. Edge 2-3	1
4	Front-side face edge Ex. Edge 2-5	1
5~10	All 6 faces	6
	Totals	10

**Table 4:** Material properties for analysis.

Components	Cushion	Box	Television	Screen
Material	EPS	Corrugated board	PPE + PS	Glass
Density (Kg/m^3^)	18–20	610	1090	1170
Poisson’s ratio	0.4	0.34	0.37	0.23

**Table 5:** Maximum stress result.

Maximum Result of Equivalent (Von-Mises) Stress, MPa							
Model	Surface (Refer Figure 3)						
	Bottom	Front	Rear	Right	Left	Edge (Right)	Edge (Left)
A	13.464	6.045	7.079	20.47	5.371	2.682	8.259
B	13.45	6.141	11.619	4.81	6.237	4.336	4.873
C	4.5547	5.3831	4.2758	4.402	6.549	4.526	5.17
D	12.58	17.08	22.65	5.558	17.88	3.197	7.526
E	2.99	12.02	8.421	6.511	1.769	1.381	4.352

## Data Availability

Not applicable.
